# Triple Head and Neck Carcinoma: Case Report and Literature Review

**DOI:** 10.7759/cureus.7082

**Published:** 2020-02-23

**Authors:** Baraa I Awad, Bashair Melibari, Nabeel Safadi, Mohammed Al-Garni, Hadi Afandi Al-Hakami

**Affiliations:** 1 Otolaryngology-Head & Neck Surgery, King Saud Bin Abdulaziz University for Health Sciences, King Abdullah International Medical Research Center, Riyadh, SAU; 2 Family Medicine, Primary Health Care Center, Makkah, SAU; 3 Radiation Oncology, King Abdulaziz Medical City, Jeddah, SAU; 4 Otolaryngology - Head and Neck Surgery, King Saud bin Abdulaziz University for Health Sciences, King Abdullah International Medical Research Center, Ministry of National Guard Health Affairs, Jeddah, SAU

**Keywords:** synchronous cancers, metachronous cancers, aero-digestive tract

## Abstract

Synchronous cancers are multiple cancers that develop within six months of the initial diagnosis while metachronous cancers are those that develop more than six months after the initial diagnosis. A combination of three cancers is seen with several patients, which leads to a bad prognosis, and that of synchronous cancers is worse than that of metachronous cancers. Herein, we describe the case of a 62-year-old woman with multiple metachronous head and neck cancers.

## Introduction

In the United States, head and neck cancers comprise approximately 4% of all other body cancers, while in developing countries, the percentage is approaching 45%, indicating that poorer socioeconomic conditions are associated with an increased incidence of head and neck cancers [1]. There are 650,000 cases diagnosed annually, among which most are in advanced stages, and approximately 350,000 patients die per year. Head and neck cancers are the fourth most common body cancer in men and the ninth most common cancer in women. The most common locations for head and neck cancers are the oral cavity and the larynx [1].

Kilciksiz defined synchronous cancers as those that develop within six months of the initial diagnosis and metachronous cancers as those that develop more than six months after the initial diagnosis [2]. Metachronous cancer refers to multiple cancers that occur after a period of time, usually in those who live relatively longer, and four types account for 50% of cancers diagnosed with second primaries: lung with bronchus, colon with rectum, breast, and prostate [3]. Several criteria define multiple head and neck cancers, including histopathological confirmation of tumor malignancy, metastases from the primary tumor, and that the tumor is separable from normal tissue [1].

Patients with existing head and neck cancers are at a greater risk of additional head and neck cancers in other areas, with either the same histopathology or different histopathology. Combinations of two primary cancers are seen in approximately 3%-5% of patients with a previous history of cancer, combinations of three cancers are seen in approximately 0.5%, and combinations of four malignant tumors are seen in approximately 0.3% [1]. The incidence of synchronous head and neck cancers is 15% and that of metachronous head and neck cancers is 4%. The median occurrence time of secondary cancer is approximately 31-43 months after initial malignancy diagnosis [1]. The incidence of secondary tumors is increased in young patients with early-stage cancer because that population survives longer than patients with advanced cancer [1]. One study reported the incidence of multiple cancers from 1978 to 1984, and in that study, 851 patients with initial squamous cell carcinomas of different head and neck regions were analyzed for the presence of secondary head and neck cancers [4]. The occurrence of secondary head and neck cancers was reported in 162 patients [4].

The most common area for multiple head and neck cancers is the aerodigestive tract with an incidence of 60%, and approximately 24% of patients initially diagnosed with laryngeal cancers develop a second primary cancer. Synchronous cancer develops in approximately 1% of patients with laryngeal cancer, while the occurrence rate of metachronous cancer is approximately 10% [1]. Patients with cancers in the oral cavity are also considered at risk of multiple head and neck cancers in the aerodigestive tract, and the incidence rate is 10%-35% [1]. Tonsillar cancer and other oropharyngeal cancers are also associated with high risks of a second primary, with reported incidence rates of up to 57% [1]. Patients with nasopharyngeal cancers have a low risk of developing a second primary [1]. Numerous cases of head and neck squamous cell carcinoma treated with neck dissection, in which metastatic papillary thyroid cancer was an incidental finding have been reported with an incidence rate of approximately 0.7%, and the second most common malignancies found incidentally are chronic lymphocytic leukemia and non-Hodgkin lymphoma, with incidence rates of approximately 0.4% [1]. Although triple head and neck cancers are extremely rare, nasopharynx, larynx, and hypopharynx cancers have a propensity for triple malignancy [5].

The etiology of multiple head and neck cancers remains unclear but several hypotheses have been suggested, including exposure to carcinogens, genetic susceptibility, immune compromisation, and initial treatments performed to address the primary cancer, causing multiple subsequent cancers [1]. Smoking and alcohol play an important role in the development of the second primary or metachronous tumors [6].

## Case presentation

A 62-year-old female non-smoker presented in July 2005 with a hard palate lesion on the left side that she had been aware of for six months and was gradually increasing in size and associated with left otalgia and left cheek pain. There was no dysphagia, no neck mass, and no weight loss or loss of appetite. Other ear, nose, and throat (ENT) history was unremarkable.

On oral cavity examination, there was a left hard palate lesion of approximately 2 × 2 cm that appeared ulcerative and bled easily. Other ENT examination results were normal. The patient underwent left incisional biopsy of the hard palate mass, which suggested adenoid cystic carcinoma, and computed tomography (CT) of the chest, abdomen, and pelvis revealed no metastases.

The patient was scheduled for left inferior maxillectomy and the insertion of a prosthesis, and the final histopathology results confirmed the diagnosis of adenoid cystic carcinoma. Postoperatively, the patient received radiotherapy of 66 GY per 33 fractions. No postoperative radiological imaging was found for this patient because the procedure and treatment were performed outside the hospital.

**Figure 1:**
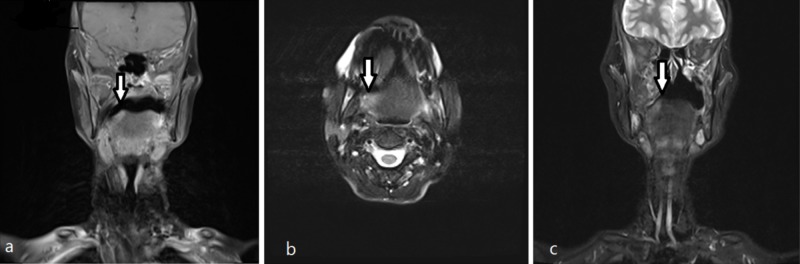
(a, b, c): Selected (a) coronal and (b) axial T2 FS show a bright lesion at the surface of the tongue on the right side confined to intrinsic muscle fibers (white arrows); it enhances in coronal T1 post-gadolinium FS image (c) with no evidence of lingual septum, root of the tongue, or bone infiltration. FS: fat saturation

The patient again presented at our ENT clinic in May 2011 with a tongue lesion on the right side, as shown in Figure 1, which she had been aware of for approximately one month without any history of dysphagia, dyspnea, or neck mass. On physical examination, the patient looked cachectic and depressed. Tongue examination revealed a tongue mass on the right side measuring 2 × 1 cm and involving the base of the tongue but not crossing the midline, which was hard on palpitation. Neck examination revealed no remarkable findings and other ENT examinations were normal. CT scanning of the neck revealed an exophytic tongue mass of approximately 2 × 2 cm on the right side. Magnetic resonance imaging (MRI) revealed a lesion signal on the surface of the tongue on the right side that was confined to the intrinsic muscle fiber, with no evidence of lingual septum, root of the tongue, or bone infiltration involvement. Lymph nodes of the neck were sub-centimetric and there was no pathological lymph node enlargement. Ear, nose, and throat structures, including the nasopharynx, oropharynx, and hypopharynx, were normal.

**Figure 2:**
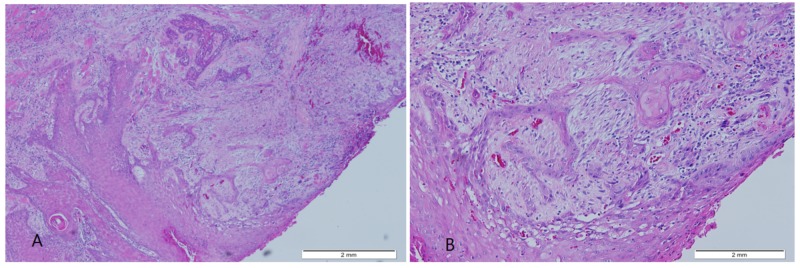
(A,B) Photomicrograph from the tongue mass (A) Squamous mucosa with underlying infiltrating islands and cords of malignant squamous cells (H&E stain; original magnification 40x). (B) The malignant cells show keratinization and mild nuclear atypia (H&E stain; original magnification 100x). H&E: hematoxylin and eosin

An incisional biopsy from the mass suggested well-differentiated squamous cell carcinoma, as shown in Figure 2. The patient underwent right partial glossectomy and right neck dissection. Histopathology confirmed the diagnosis of well-differentiated squamous cell carcinoma. There were 35 lymph nodes, all without metastases. The depth of the tumor was 5 mm, it had negative margins, and no lymphatic invasion was apparent. She did not receive any postoperative treatment.

**Figure 3:**
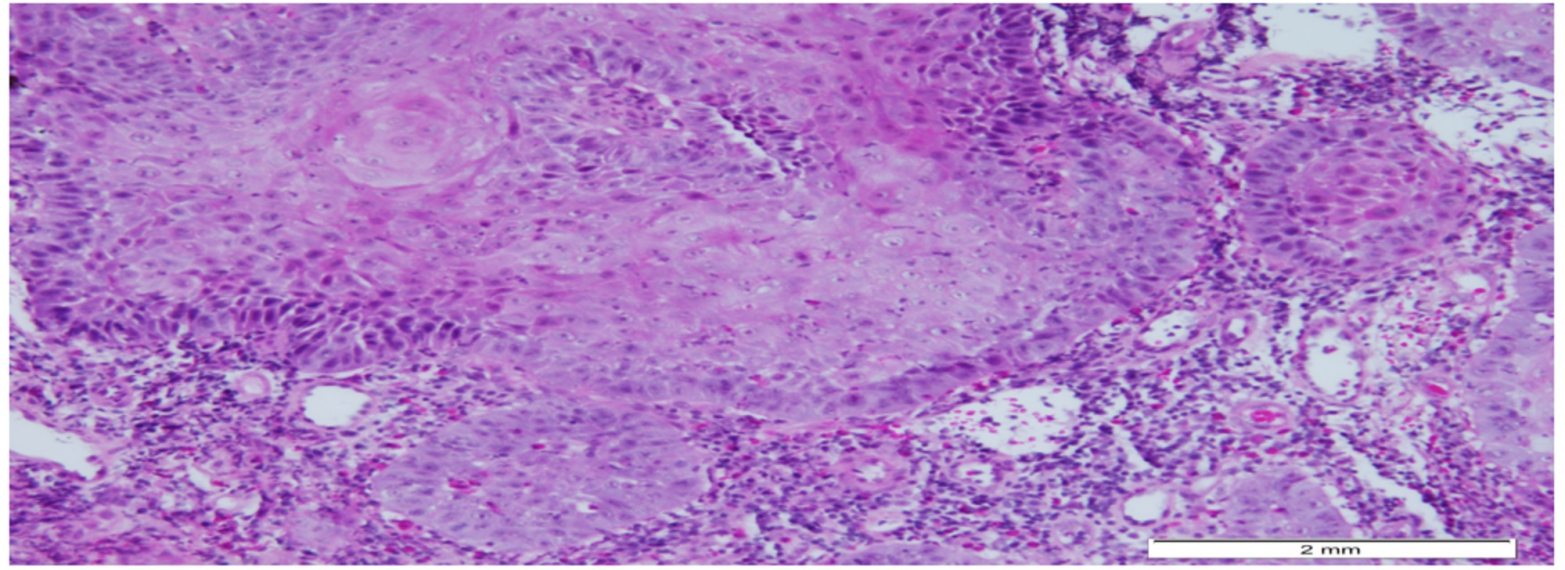
Photomicrographs from the nasopharyngeal mass shows Infiltrating malignant squamous cells in islands (H&E stain; original magnification 100x) H&E: hematoxylin and eosin

**Figure 4:**
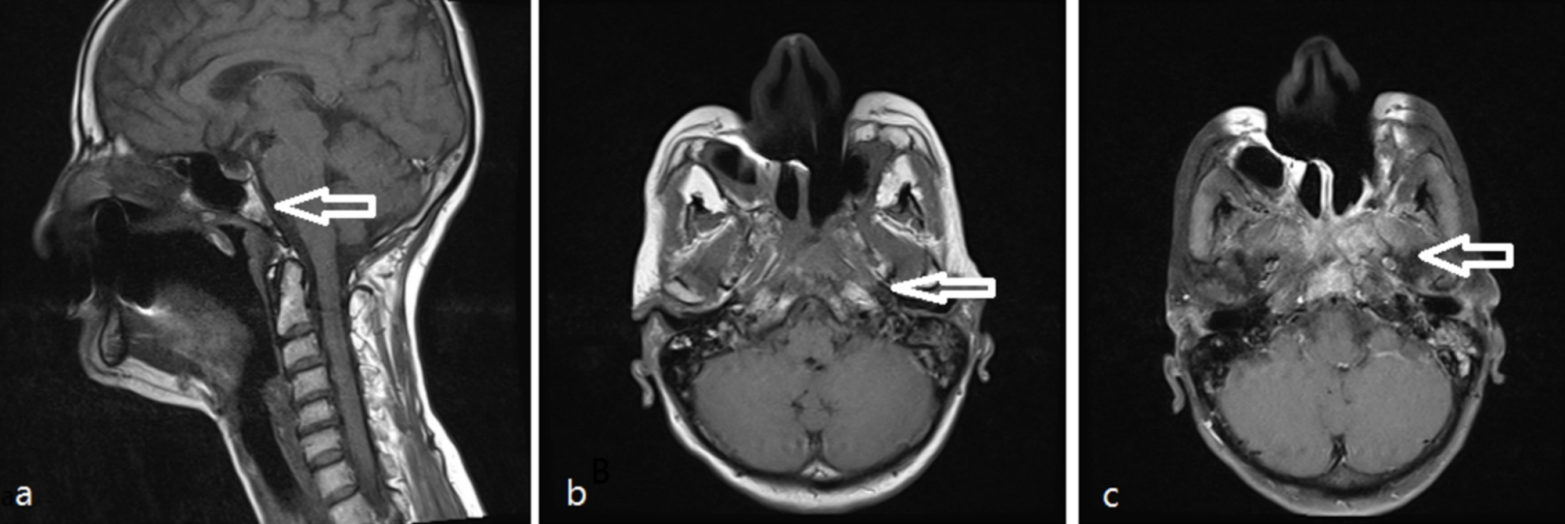
(a, b, c) Selected sagittal (a) and axial (b) T1 WI shows left nasopharyngeal mass (white arrows) that infiltrates the clivus. (c) The mass enhances in axial T1 post-gadolinium image, note the retained secretion in left mastoid air cells.

In April 2017, the patient again presented to the ENT clinic complaining of left-side hearing loss. On examination, there was otitis media, with effusion on the left side. Nasal scoop examination revealed a nasopharyngeal mass on the left side. Biopsy suggested well-differentiated skeletonizing squamous cell carcinoma, as shown in Figure 3. CT scanning revealed a nasopharyngeal soft tissue mass involving the superolateral nasopharyngeal wall on the left side, as shown in Figure 4. Other surrounding structures were normal but there was an enlarged level 1B lymph node on the left side measuring 0.9 cm and an enlarged level 2 lymph node on the right side measuring 1.8 cm. MRI depicted an ill-defined infiltrative mass of the nasopharynx involving both sides but, predominantly, the left side with extension to the left parapharyngeal fat, retropharyngeal muscle, and clivus consisting of stage T3 tumor. No obvious perineural split was apparent.

The patient was diagnosed with nasopharyngeal carcinoma and received radiation therapy consisting of 72 GY per 60 fractions b.i.d. Post-treatment MRI revealed changes on the left side of the nasopharynx associated with residual inflammatory changes extending to the left side of the sphenoid bone. The study was limited because there was an artifact.

During her regular follow-up, the patient presented to the dental clinic because she had developed a fracture of the upper jaw. Examination revealed an enlarged upper neck lymph node of approximately 1 × 2 cm on the right side. CT scanning of the neck revealed the recurrence of the previously observed nasopharyngeal asymmetry with fat infiltration noted in the left nasopharyngeal space involving the lateral pterygoid muscle with subtle superior extension to the left pterygopalatine fossa reaching the lower fibers of the left temporalis muscle. A lytic lesion was noted within the odontoid process with tiny air pockets on its deeper fascia.

With regard to the neck status, recurrences of the previously observed sub-centric pathological lymph nodes at level 1A, 1B bilateral, and 2A and 2B, predominantly on the left side, were identified via post-radiotherapy imaging, but they were not enlarged according to cross-section criteria. The aforementioned findings at the left nasopharynx skull base odontoid process were highly suggestive of local recurrence. Nasopharyngoscopy revealed an irregular infiltrative mass involving the whole nasopharynx. The patient was diagnosed as having recurrent nasopharyngeal cancer and was considered a palliative case. She was classified as “no code” due to her poor medical condition and treatment-resistant disease.

## Discussion

Patients with head and neck squamous cell carcinoma are more likely to develop a second primary malignancy in the upper aerodigestive tract. Although small case series have included patients with thyroid malignancy presenting with laryngeal squamous cell carcinoma, lymphoid malignancies are rarely associated with the development of squamous cell carcinoma [5].

Head and neck cancers are considered some of the most challenging cancers to manage in surgical and oncological practice, and the process is made more complex by the scant evidence base available to support management options [1]. Patients with recurrent head and neck cancers are usually considered to have poor prognoses [1]. Prognostic factors vary according to the presence or absence of metastases as well as the site and size of tumors that may metastasize to lymph nodes of the neck [4]. Because the incidence of head and neck cancer is increasing and recurrence can be fatal, screening programs and/or chemo-prevention is required, as such measures facilitate early diagnoses and improved outcomes [4].

While triple head and neck cancer is very rare, the risk of it increases with age and the effects of the first tumor on the second primary and vice versa remain unclear. The second primary tumor tends to be more aggressive, and the prognosis of synchronous tumors is extremely poor compared to that of metachronous malignancies [1,5]. In Western literature, second primary malignancy (SPM) tends to be the leading cause of mortality after head and neck squamous cell carcinoma [1]. The current patient underwent multimodal management and her response to chemoradiotherapy for the third cancer was poor. This may have been related to poor response or strong resistance, which developed due to repeated therapy.

## Conclusions

Triple head and neck cancer, which most commonly occurs in the aerodigestive tract, is an extremely rare condition that is associated with a high chance of morbidity and mortality. The incidence increases with age and the screening program is required to establish an early diagnosis.
